# The Sam-Sam interaction between Ship2 and the EphA2 receptor: design and analysis of peptide inhibitors

**DOI:** 10.1038/s41598-017-17684-5

**Published:** 2017-12-12

**Authors:** Flavia Anna Mercurio, Concetta Di Natale, Luciano Pirone, Roberta Iannitti, Daniela Marasco, Emilia Maria Pedone, Rosanna Palumbo, Marilisa Leone

**Affiliations:** 1 Institute of Biostructures and Bioimaging (IBB), CNR, via Mezzocannone 16, 80134 Naples, Italy; 2 Department of Pharmacy, Research Centre on Bioactive Peptides (CIRPeB), University of Naples “Federico II”, Via Mezzocannone 16, 80134 Naples, Italy

## Abstract

The lipid phosphatase Ship2 represents a drug discovery target for the treatment of different diseases, including cancer. Its C-terminal sterile alpha motif domain (Ship2-Sam) associates with the Sam domain from the EphA2 receptor (EphA2-Sam). This interaction is expected to mainly induce pro-oncogenic effects in cells therefore, inhibition of the Ship2-Sam/EphA2-Sam complex may represent an innovative route to discover anti-cancer therapeutics. In the present work, we designed and analyzed several peptide sequences encompassing the interaction interface of EphA2-Sam for Ship2-Sam. Peptide conformational analyses and interaction assays with Ship2-Sam conducted through diverse techniques (CD, NMR, SPR and MST), identified a positively charged penta-amino acid native motif in EphA2-Sam, that once repeated three times in tandem, binds Ship2-Sam. NMR experiments show that the peptide targets the negatively charged binding site of Ship2-Sam for EphA2-Sam. Preliminary *in vitro* cell-based assays indicate that -at 50 µM concentration- it induces necrosis of PC-3 prostate cancer cells with more cytotoxic effect on cancer cells than on normal dermal fibroblasts. This work represents a pioneering study that opens further opportunities for the development of inhibitors of the Ship2-Sam/EphA2-Sam complex for therapeutic applications.

## Introduction

Several signaling proteins are engaged to the plasma membrane by the lipid second messenger PIP3 (phosphatidylinositol 3,4,5-triphosphate) whose intracellular levels are regulated by phosphoinositide phosphatases. Among them, Ship2 (phosphatidylinositol 3,4,5-triphosphate 5-phosphatase 2) catalyses the dephosphorylation of PIP3 in position 5 to generate phosphatidylinositol (3, 4) P2 and thus, downregulates different processes that are activated by PI3K (Phosphatidyl-Inositol 3 Kinase)^1,2^. In addition to this enzymatic activity, a prominent feature of Ship2 is the presence within its primary sequence of several regions able to mediate protein-protein interactions. In details, Ship2 includes from the N- to the C-termini a SH2 (Src homology 2) domain, followed by the catalytic 5-phosphatase domain, -NPXY- motifs, that generally recognize phosphotyrosine binding (PTB) domains, a proline-rich domain (PRD) with consensus sequences for SH3 modules and a sterile alpha-motif (Sam) domain^3^.

Ship2 interacts with several other proteins and acts in different processes like receptor internalization, cell spreading and adhesion, actin cytoskeletal reorganization^4^.

Ship2 is a well known target in drug discovery for type 2 diabetes as it modulates insulin sensitivity and obesity^5^. Nevertheless, Ship2 has been linked to other diseases such as neurodegenerative pathologies, atherosclerosis, as well as cancer^4^. The role of Ship2 in cancer needs to be further elucidated and is controversial. Ship2 is over-expressed in colorectal cancer where it indicates poor survival^6^. Ship2 inhibition and consequent Akt activation in gastric cancer cells contribute to improved tumorigenesis and proliferation^7^. Interestingly, Ship2 modulates EGFR (Epidermal Growth Factor Receptor) signaling: down-regulation of Ship2 in breast cancer cell lines improves EGFR internalization and degradation and arrests cell proliferation^8^.

Ship2 is also an inhibitor of EphA2 receptor endocytosis^9^; EphA2 is a tyrosine kinase receptor that plays a complex role in cancer and is a known target in anticancer drug discovery^10^.

To achieve modulation of receptor endocytosis, Ship2 needs to associate with EphA2 through a heterotypic Sam-Sam domain interaction^9^.

**Figure 1:**
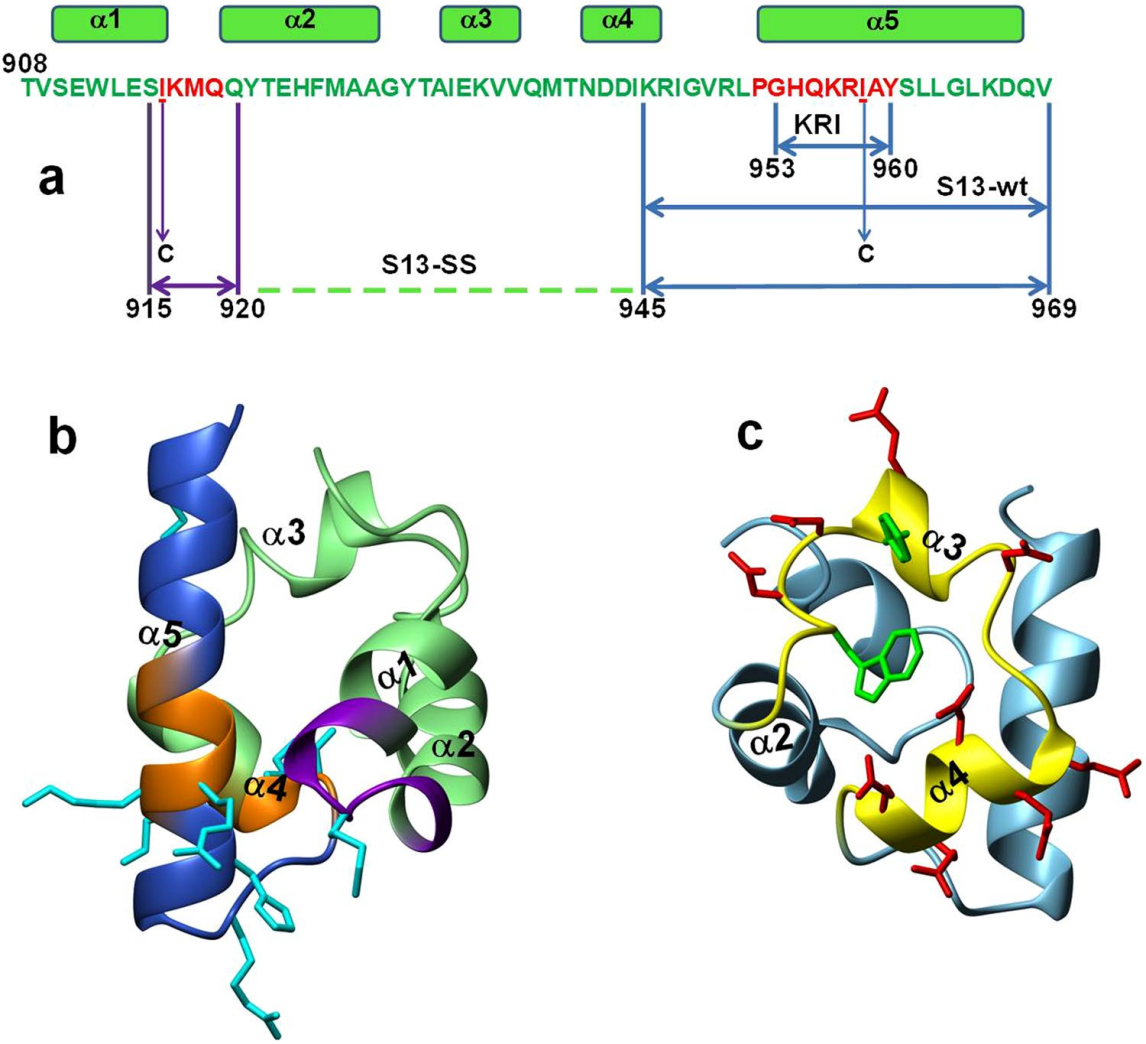
(**a**) Amino acid sequence of EphA2-Sam (UniprotKB entry P29317 EPHA2_HUMAN) with secondary structure elements indicated on top (from first conformer in pdb entry 2E8N by Goroncy *et al*., 2007 Structural Genomics/Proteomics Initiative). Residues in the α1α2 loop and in the α5 helix belong to the EH region and are colored red. The analyzed peptide segments are delineated by arrows. (**b**) NMR solution structure of EphA2-Sam (first conformer, pdb entry 2E8N) in a ribbon representation: regions encompassing the S13-wt peptide are colored blue with the “KRI” and “KRIAY” sequences highlighted in orange; the additional fragment contributing to the S13-SS peptide is colored violet. The side chains of positively charged residues are shown in cyan. (**c**) NMR solution structure of Ship2-Sam (pdb entry 2K4P structure n.1^11^). The ML interface is colored yellow and side chains of acidic and aromatic amino acids are reported in red and green respectively.

The 3D structures of the Sam domains of EphA2 (EphA2-Sam) and Ship2 (Ship2-Sam)^11^ consist of a canonical five helix bundle (Fig. 1). EphA2-Sam and Ship2-Sam bind each other with a dissociation constant in the low micromolar range and a 1:1 stoichiometry^11,12^. The two proteins adopt the ML (Mid-Loop)/EH (End-Helix) interaction model characteristic of Sam/Sam associations^11,12^. The Ship2-Sam/EphA2-Sam complex is highly stabilized by electrostatic contacts between the negatively charged central region of Ship2-Sam (ML site) and the positively charged interface of EphA2-Sam, that includes the C-terminal α5 helix and the adjacent loop (EH site)^11^ (Fig. 1). Moreover, the Sam-Sam complex is highly dynamic and able to sample different conformational states^13,14^.

In cancer cells the interaction between Ship2-Sam and EphA2-Sam is expected to produce mainly pro-oncogenic effects, because Ship2 enhances the ligand-independent pro-migratory function of EphA2 and decreases its ligand-dependent tumor suppressor roles^12^. These findings let speculate that antagonizing the heterotypic Ship2-Sam/EphA2-Sam interaction, by lowering EphA2 pro-oncogenic signaling, may constitute a novel approach for anticancer therapeutics.

With this in mind, we focused on peptide fragments from the EH interface of EphA2-Sam and investigated their conformational properties by means of CD and NMR experiments. Our work highlights that in aqueous solution these fragments do not preserve a native-like fold and are unable to relevantly bind Ship2-Sam in SPR (Surface Plasmon Resonance) and NMR (Nuclear Magnetic Resonance) interaction assays. Nevertheless, these studies led to the identification of a positively charged penta-amino acid motif that, when repeated three times in tandem, binds the ML interface of Ship2-Sam with a dissociation constant in the high micromolar range.

Preliminary *in vitro* cell based assays demonstrate that the peptide is more cytotoxic to prostate cancer cells (PC-3) than to normal human dermal fibroblasts (NHDF).

Our work sheds further light on possible routes to target Sam-Sam interactions mediated by EphA2 and opens a window of opportunities for the design of novel compounds with different therapeutic applications.

## Results and Discussion

### Peptide Design

To identify peptide ligands of Ship2-Sam, we analyzed isolated EphA2-Sam regions in or close to the EH interface^11^ (Fig. 1). Thus, the linear S13-wt peptide, corresponding to the 945–969 fragment from EphA2-Sam and, including the C-terminal α5 helix, the α4α5 loop and partially the C-terminal end of α4 helix (Fig. 1a,b), was first investigated. The S13-SS peptide was next conceived to better mimic the discontinuous epitope characterizing the EH interface (Fig. 1a). S13-SS includes, in addition to identical S13-wt regions, an EphA2-Sam portion covering mostly the α1α2 loop (a.a. 915–920). Moreover, two Isoleucine residues (i.e., Ile916 and Ile958) in the native sequence were mutated to cysteines to allow disulphide linkage of the two segments (915–920 and 945–969) (Fig. 1a,b and Supplemental Table S1).

Since the ML Interface in Ship2-Sam is negatively charged and also contains both a tryptophan and a phenylalanine^11^ (Fig. 1c) we chose to analyze alone the short peptide motif -GHQKRIAY- (hereof named “KRI” peptide, Table S1), that is present at the N-terminal side of α5 helix in EphA2-Sam (Fig. 1a,b). It has been speculated that an intermolecular H-bond involving the backbone amide proton of the Gly residue within the “KRI” peptide sequence, may be crucial for the association between Sam domains^15,16^. Interestingly, the KRI peptide includes both aromatic residues and a stretch of positively charged amino acids which could all interact with the ML interface of Ship2-Sam (Fig. 1c). To maximize potential electrostatic and aromatic interactions with Ship2-Sam, two additional peptides containing the -KRIAY- penta-amino acid motif, repeated two (=(KRI)_2_ peptide) and three (=(KRI)_3_ peptide) times in tandem were studied as well (Fig. 1a and Supplemental Table S1).

### Conformational analysis by CD

Peptide conformational studies were first performed in solution by far-UV CD spectroscopy. CD spectra in aqueous buffer clearly indicate for all peptides a prevalent random coil content for the minimum at ~200 nm (Supplemental Fig. S1). However, S13-wt and (KRI)_3_ show a certain propensity to assume an α-helical conformation as indicated by TFE (2,2,2-trifluoroethanol) titration (Supplemental Fig. S2).

### Structural insights by NMR spectroscopy

Additional conformational studies were carried out by NMR spectroscopy. In aqueous solution all peptides appear unstructured in agreement with CD data. Almost complete proton resonance assignments in PBS were obtained for S13-wt (Supplemental Table S2). However, S13-wt and (KRI)_3_ could be better studied in presence of TFE (2,2,2-trifluoroethanol) (Supplemental Figs S3 and S4, Tables S3, S4 and S5). TFE can reveal the intrinsic tendency of an amino-acid sequence to assume a specific secondary structure that may characterize its conformation when interacting with membranes or different peptides/proteins^17,18^. Our recent work on several fragments from different Sam domains (Ship2^19^ and the adaptor protein Odin^20^) further shows, in agreement with earliest studies^21^, that native-like helices are well reproduced in solution containing TFE. However, propagation of helical content to the more disordered protein regions may occur at high TFE concentration^20^.

The comparison of Hα chemical shifts deviations from random coil values in PBS and in TFE solution clearly indicates for S13-wt an increase in helical content, with the percentage of helical population, estimated by chemical shifts, raising from 10% in PBS to 29% in presence of TFE^22^ (60% v/v) (Supplemental Fig. S3a). NMR studies in TFE reveal a completely native-like fold for the S13-wt peptide (Supplemental Fig. S3b,c and Table S4).

Similarly, structure calculations performed for the (KRI)_3_ peptide in presence of 50% TFE indicate the presence of an α-helix extending through the whole peptide sequence (Supplemental Fig. S4 and Table S5).

### Binding studies with EphA2-Sam

#### SPR and MST experiments

**Figure 2:**
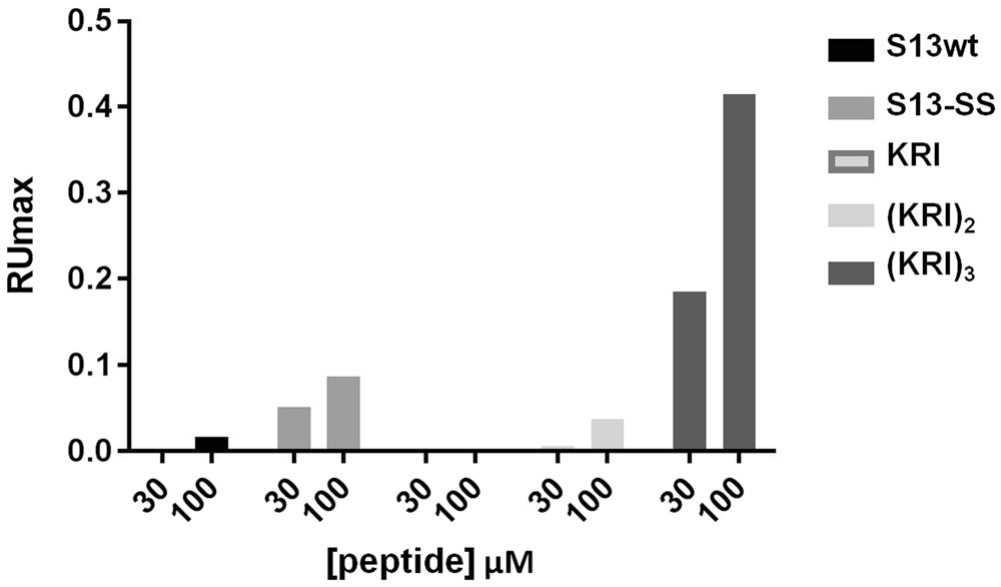
SPR experiments to analyze binding of different peptides to immobilized Ship2-Sam. RU_max_ values derive from sensorgrams recorded at two different concentrations (30 and 100 µM) and signals are normalized for the molecular weight of each peptide.

**Figure 3:**
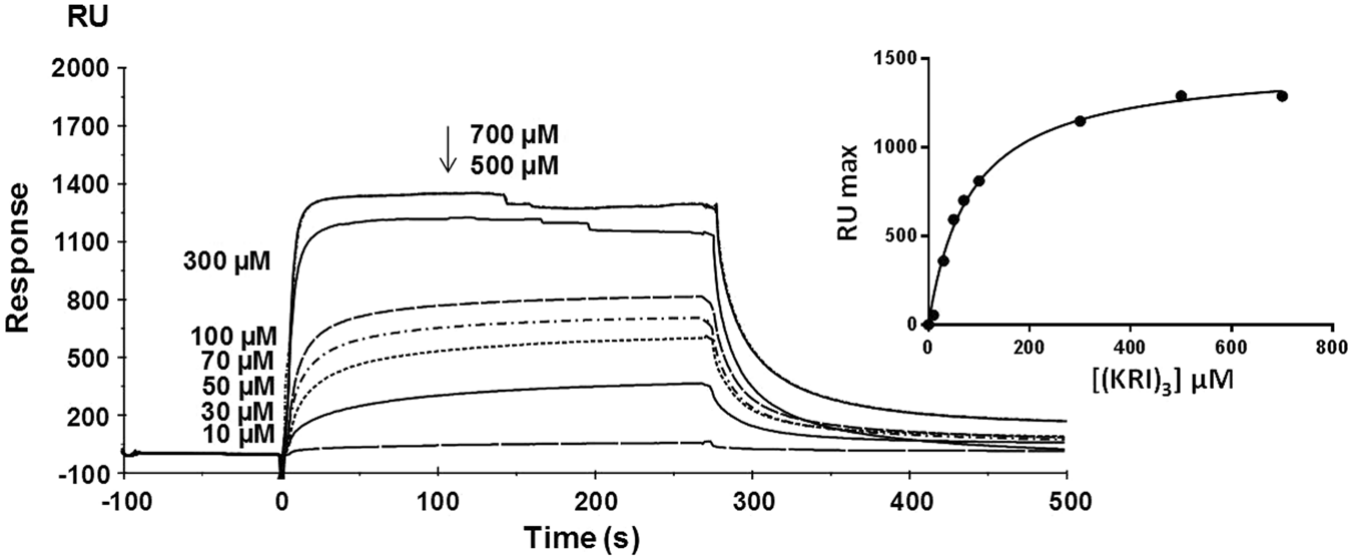
Interaction between (KRI)_3_ and Ship2-Sam monitored by SPR experiments. Sensorgrams were recorded at increasing peptide concentrations and Ship2-Sam was immobilized on the microchip surface. The plot of RU_max_ values as function of peptide (KRI)_3_ concentration is shown in the inset.

In order to investigate the interaction with Ship2-Sam, all peptides were analysed by SPR experiments (Figs 2, 3 and S5).

Histograms of Response Unit (RUmax) values, evaluated at 30 and 100 µM concentrations, show that the KRI and (KRI)_2_ peptides are not able to bind Ship2-Sam while (KRI)_3_ and S13-SS generate significant dose-response signal variations (Fig. 2). SPR sensorgrams recorded for S13-SS in a more wide concentration range, indicate that this peptide does not reach saturation (Supplemental Fig. S5b). Sensorgrams of the (KRI)_3_ peptide, recorded at concentrations of 500 and 700 µM, appear quite noisy suggesting partial insolubility at the Ship2-Sam interface (Fig. 3). However, (KRI)_3_ curves were fitted according to a single binding site model with 1:1 stoichiometry and provided a K_D_ value of 100 µM, that is in agreement with the one measured by the binding isotherm K_D_ = 83 ± 8 µM (Table 1 and Fig. 3).

**Table 1:** Dissociation constants measured in SPR experiments for binding of (KRI)_3_ to Ship2-Sam.

	SPR kinetic			SPR binding isotherm
**(KRI)**_**3**_	k_on_ (M^−1^s^−1^)	k_off_ (s^−1^ × 10^−3^)	K_D_ (µM)	K_D_ (µM)
	1100	0.110	100	83

The interaction between the (KRI)_3_ peptide and Ship2-Sam was further investigated by MST (Microscale Thermophoresis) experiments (Supplemental Fig. S6)^23^. MST studies clearly show a protein/peptide interaction but, they do not allow an accurate K_D_ measurement due to precipitate formation at the highest (KRI)_3_ peptide concentrations (Supplemental Fig. S6).

#### NMR and CD assays

**Figure 4:**
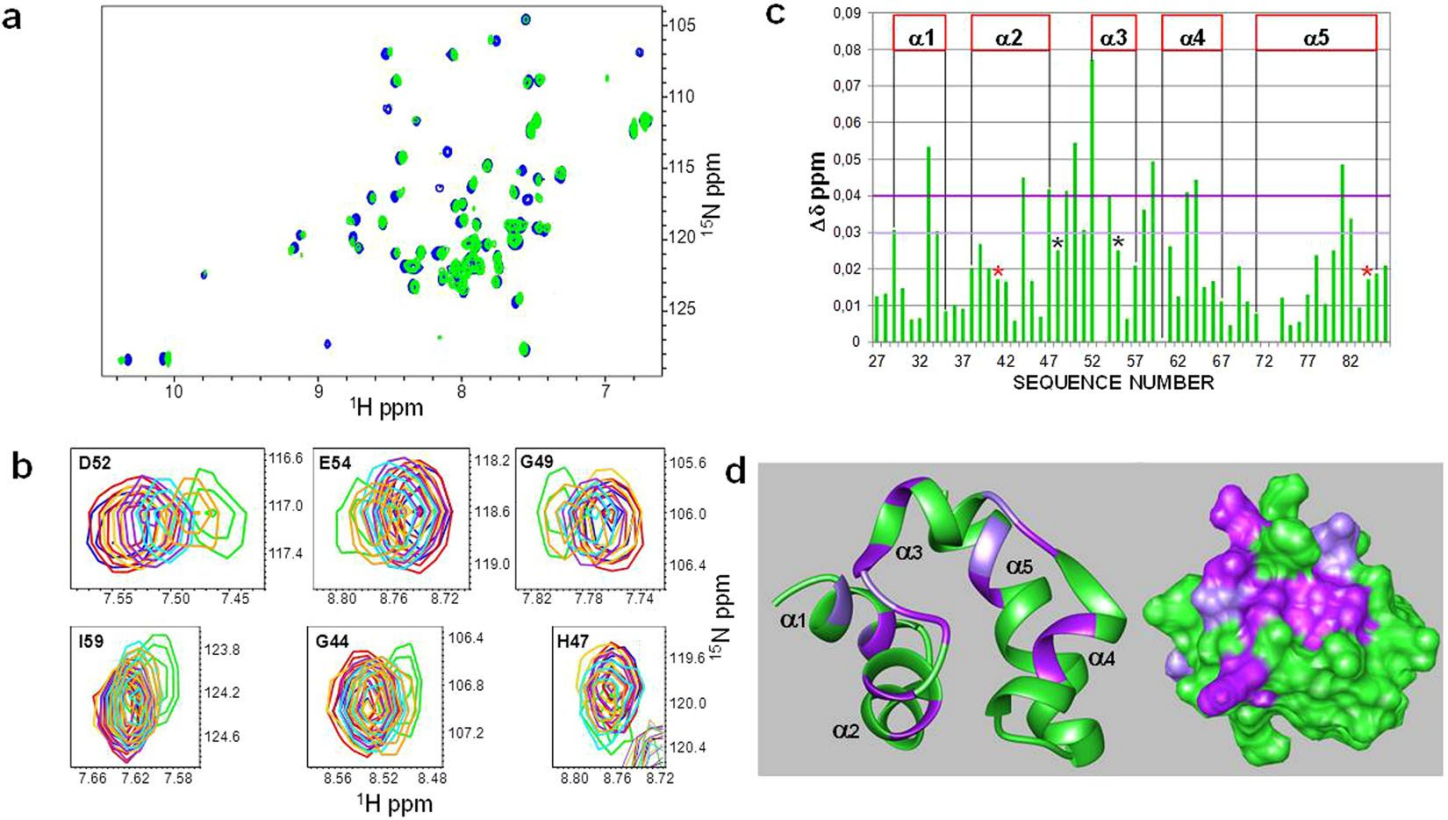
(**a**) Superposition of [^1^H-^15^N] HSQC spectra of Ship2-Sam in its apo form (40 μM concentration -blue) and after addition of the (KRI)_3_ peptide (2.0 mM concentration -green). (**b**) A few Ship2-Sam residues, experiencing major chemical shift variations during titration with the (KRI)_3_ peptide, are highlighted. The different panels show the overlays of [^1^H-^15^N] HSQC spectra of Ship2-Sam alone (40 μM concentration -blue) and in presence of increasing amounts of (KRI)_3_ (80 μM -red; 152 μM -gold; 300 μM -purple; 586 μM -cyan; 1.1 mM -orange; 2.0 mM -green). (**c**) Graph reporting normalized chemical shift deviations (Δδ = [(ΔH_N_)^2^ + (0.17 * Δ^15^N)^2^]^1/2^)^55^ *versus* residue number. Δδ values were set equal to 0 for P72 as well as L53, T60 whose peaks disappear during titration. Δδ values are ambiguous for Y41 and L84 (red stars) and for N48 and F55 (black stars) due to spectral overlaps. (**d**) Residues with normalized chemical shifts deviations (Δδ values) ≥ 0.04 ppm (i.e., L33, G44, H47, G49, W50, D52, E54, I59, D63, L64, T81) or comprised between 0.03 and 0.04 ppm (i.e., M29, R34, D51, D58, L82) are colored in dark and light violet respectively on the 3D solution structure of Ship2-Sam (pdb entry 2K4P, conformer number 1) in its ribbon (left) and surface (right) representations.

To further analyze the interaction between Ship2-Sam and each peptide, an NMR-based screening was conducted by means of chemical shift perturbation experiments with HSQC spectra^24^ and ^15^N labeled Ship2-Sam (Figs 4 and S7). These experiments appear in perfect agreement with SPR data and show more significant chemical shifts variations in the spectrum of Ship2-Sam only upon addition of the (KRI)_3_ peptide (Fig. 4). Moreover, as already observed in MST assays, at the highest peptide concentrations (values > 300 µM) precipitation occurs with loss of protein signal (Fig. 4a,b) and this avoids reaching saturating conditions and achieving accurate K_D_ evaluation. Nevertheless, we evaluated CSP (Chemical Shifts Perturbations) induced in the protein spectrum by the peptide and mapped them on the 3D solution structure of Ship2-Sam (Fig. 4c,d). Interestingly, most of the changes affects the highly negatively charged ML region of Ship2-Sam and the external close regions (such as the C-terminal side of α5 helix), which may undergo “indirect” conformational movements. Indeed the pattern of variations closely resembles the ones observed for the interaction between Ship2-Sam and EphA2-Sam^11^ (Fig. 4d).

**Figure 5:**
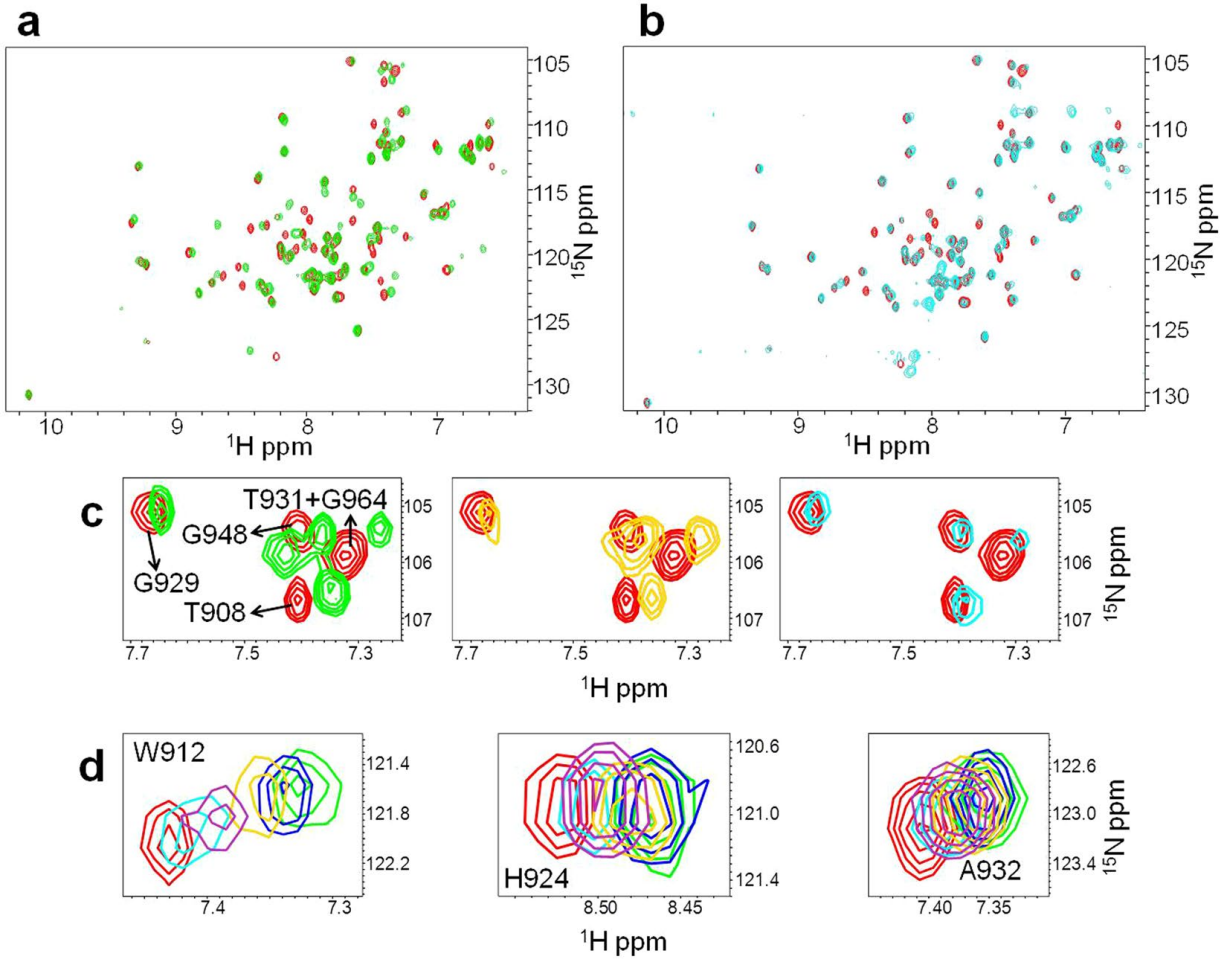
(**a,b**) Superposition of [^1^H-^15^N] HSQC spectra of ^15^N labeled EphA2-Sam (25 μM, red), bound to unlabeled Ship2-Sam (80 μM, green) (**a**), and after addition of (KRI)_3_ (7 mM concentration) to the complex (cyan) (**b**). (**c**) Expansion of the Gly-Thr region in HSQC spectra of ^15^N labeled EphA2-Sam in the free form (red in all panels), in presence of unlabeled Ship2-Sam (green, left panel), and after addition of (KRI)_3_ (2.2 mM concentration -gold, middle panel and 7 mM concentration -cyan, right panel). (**d**) Overlays of [^1^H-^15^N] HSQC spectra showing results of the displacement experiment for a few residues (red: ^15^N EphA2-Sam apo; green: ^15^N EphA2-Sam/Ship2-Sam complex; blue: ^15^N EphA2-Sam/Ship2-Sam + (KRI)_3_ at 1.1 mM concentration; gold: ^15^N EphA2-Sam/Ship2-Sam + (KRI)_3_ at 2.2 mM concentration; purple: ^15^N EphA2-Sam/Ship2-Sam + (KRI)_3_ at 4.9 mM concentration; cyan: ^15^N EphA2-Sam/Ship2-Sam + (KRI)_3_ at 7 mM concentration).

To further demonstrate that EphA2-Sam and the (KRI)_3_ peptide are (completely or partially) targeting the same Ship2-Sam regions, a displacement experiment^25,26^ was conducted (Fig. 5). Upon addition of unlabelled Ship2-Sam to ^15^N-labeled EphA2-Sam, the [^1^H, ^15^N] HSQC spectrum undergoes several changes canonical for the Sam-Sam association (Fig. 5a)^11^. By further adding increasing amounts of the (KRI)_3_ peptide, many peaks within the spectrum of the complex restore the characteristic position of the unbound EphA2-Sam form (Fig. 5b,c,d). However, despite the presence of a large excess of (KRI)_3_, displacement of Ship2-Sam is only partial (Fig. 5b). These results are likely due to the weaker binding affinity of Ship2-Sam for the (KRI)_3_ peptide (Table 1) with respect to EphA2-Sam (the K_D_ value for the Ship2-Sam/EphA2-Sam complex is 0.75 ± 0.12 µM in ITC experiments^11^). Interestingly, a patch of negatively charged residues is located in proximity of the α2 helix in Ship2-Sam (Supplemental Fig. S8). We cannot exclude that at the highest peptide concentrations, (KRI)_3_ may also target this area, that is adjacent to the ML interface, and this would reduce the exposed polar surface causing precipitation of the peptide/protein complex.

A transferred-NOESY experiment^27^ was conducted and failed to evidence formation of a rigid defined conformation in the (KRI)_3_ peptide upon binding to Ship2-Sam (Supplemental Fig. S9). Since the relatively low molecular weight of Ship2-Sam might have avoided observing peptide folding by NMR upon association with the protein^27^, CD spectra were as well recorded for (KRI)_3_ in the presence of different equivalents of Ship2-Sam (Supplemental Fig. S10). The overlay of CD spectra indicates a certain increase of order upon complex formation but, in agreement with NMR data, allows excluding a (KRI)_3_ bound conformation provided with a fully defined secondary structure (Supplemental Fig. S10).

### Effects of (KRI)_3_ peptide in cancer cells

**Figure 6:**
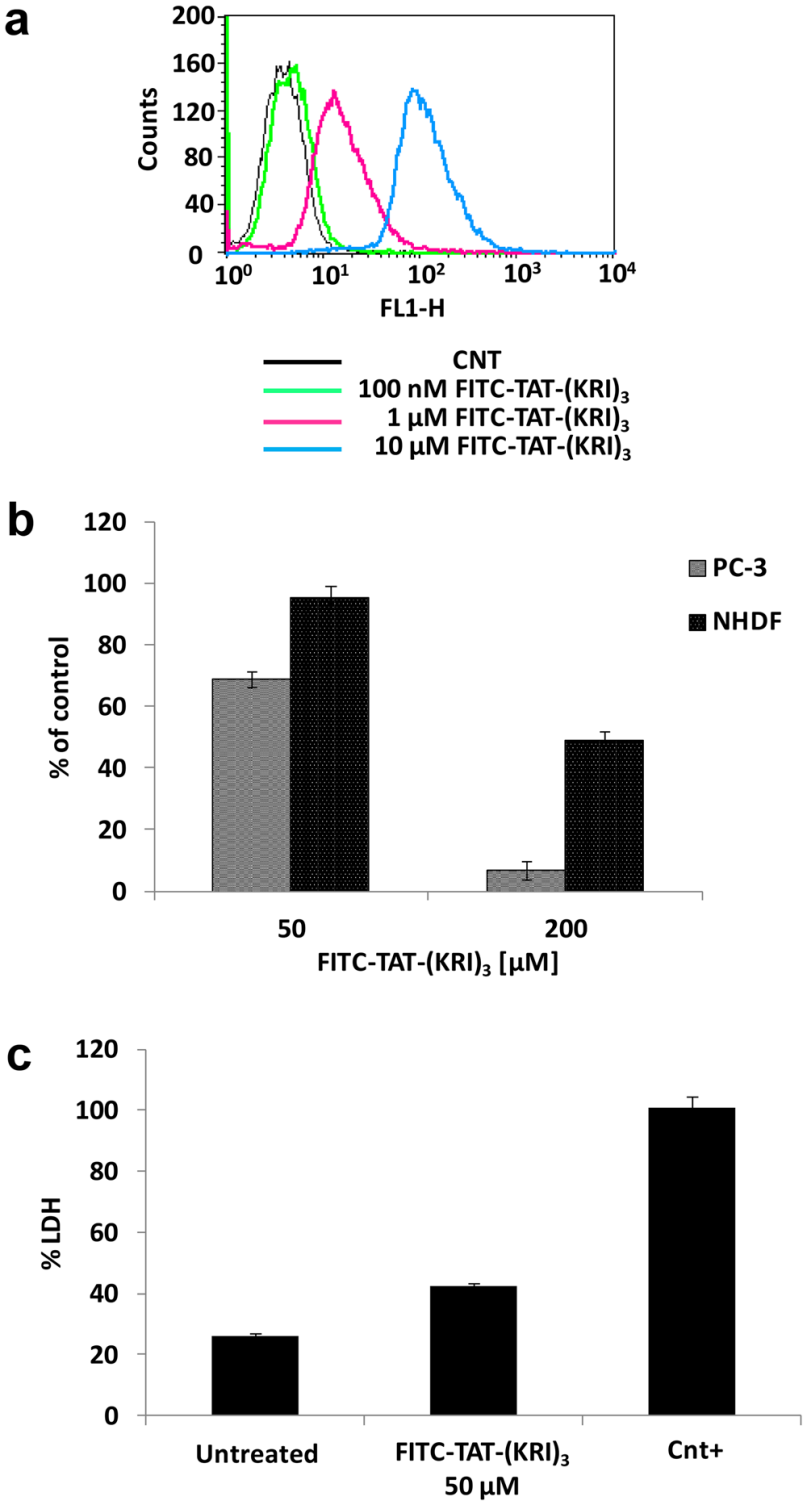
Cellular uptake and *in vitro* cytotoxicity of FITC-TAT-(KRI)_3_. (**a**) Internalization of the peptide was detected by flow cytometric analysis on permeabilized PC-3 cells after incubation with different amounts of FITC-TAT-(KRI)_3_ for 4 hours. Untreated cells were used as negative control (CNT). The histogram was obtained from a single experiment and was representative of three independent measurements. (**b**) The effect of FITC-TAT-(KRI)_3_ on cell viability was assessed on PC-3 and NHDF cells. Cancer and normal cells were incubated with peptide at 50 and 200 μM concentrations, after 4 hours the crystal violet assay was performed. Data are expressed as percentage of negative control. (**c**) The cytotoxic effect of 50 μM FITC-TAT-(KRI)_3_ on PC-3 cells was evaluated by measuring the LDH release in culture medium. PC-3 cells treated with 1% Triton X-100 were used as positive control (Cnt+). The percentage of released LDH was calculated as indicated in the Materials and Methods. Each value is the mean ± SD of three independent experiments that were each performed in quadruplicate.

After conjugating the peptide (KRI)_3_ to a cell penetrating sequence (i.e., fragment 48–60 from the HIV TAT sequence (Supplemental  Table S1)^28,29^), its effects in a cellular context were evaluated (Fig. 6). Upon assessing cellular uptake (Fig. 6a), cell viability was first investigated in absence and presence of the FITC (Fluorescein-Isothiocyanate)-TAT-(KRI)_3_ peptide in the carcinoma prostate cancer cell line PC-3 (Fig. 6b). PC-3 cells were chosen due to their high endogenous levels of EphA2^30,31^ and Ship2^32^. After treatment with the FITC-TAT-(KRI)_3_ peptide (50 µM and 200 µM concentrations) a reduction of cell viability of approximately 30% and 90% respectively, compared to the untreated cells, was observed (Fig. 6b).

NMR analysis of the FITC-TAT-(KRI)_3_ peptide clearly indicates that the conformational freedom of the (KRI)_3_ peptide is not influenced by the presence of the FITC-TAT unit (Supplemental Fig. S11). To exclude that the reduction of cell viability could be merely linked to the FITC or the TAT sequence, experiments were conducted with FITC-TAT-Pep1 (Supplemental Table S1 and Fig. S12). We previously described Pep1 as a weak EphA2-Sam ligand with an amino acid sequence encompassing the central regions of the first Sam domain of Odin^20^. At 250 µM concentration FITC-TAT-Pep1 induces only a small decrease of PC-3 cells viability (Supplemental Fig. S12) thus indicating that cytotoxicity of FITC-TAT-(KRI)_3_ is specifically linked to the (KRI)_3_ sequence.

Moreover, the activity of FITC-TAT-(KRI)_3_ was also analyzed in Normal Human Dermal Fibroblasts (NHDF). The FITC-TAT-(KRI)_3_ peptide, at the highest tested concentration (i.e., 200 µM), has less cytotoxic activity in NHDF than in PC-3 cells (Fig. 6b).

The FITC-TAT-(KRI)_3_ peptide induces necrosis of PC-3 cells by damaging plasma membrane and causing LDH (lactate dehydrogenase) leakage (Fig. 6c)^33^.

It has recently been pointed out that pro-necrotic peptides may represent a different route to overcome the limits of pro-apoptotic anti-cancer compounds and that their specificity towards cancer cells can be highly increased by fusion to tumor-homing motifs^34^.

To get further insights into the biological function of the (KRI)_3_ peptide, its “KRIAY” motif was searched against a database of bioactive peptides (i.e., the EROP (Endogenous Regulatory OligoPeptide knowledgebase)-Moscow at http://erop.inbi.ras.ru/)^35^ but no hits could be retrieved. Nevertheless, a similar search of the “KRI” sequence produced several positive results including antimicrobial and antibacterial peptides^36,37^. On the same time, no hits could be retrieved by searching the sequence “KRIAY” against a database of anticancer peptides and proteins (http://crdd.osdd.net/raghava/cancerppd/) whereas the search of the “KRI” sequence produced several results among antibacterial and antimicrobial peptides with anticancer activity. In major details, the “KRI” three amino acid motif is found in the peptide Cecropin P1, that has biological activity against diverse bacteria as well as mammalian leukemias and lymphomas^38^; in the peptide LL-37^39,40^, that reduces cell viability in many cancer cell lines including ovarian (OVRCAR-3), breast (MCF-7) and prostate cancer cell lines (LNCaP); finally in the peptide NK-11 that is active against skin cancer^41^.

## Conclusions

Targeting the Ship2-Sam/EphA2-Sam complex with peptide antagonists may represent an alternative approach for the discovery of anticancer therapeutics. Thus, we analyzed isolated fragments of EphA2-Sam encompassing its binding site for Ship2-Sam (EH interface) and the close loop regions. We adopted a multidisciplinary methodology involving conformational analysis by CD and NMR spectroscopies, binding assays by NMR, SPR and MST techniques, *in vitro* cell-based experiments.

Structural studies point out that isolated EphA2-Sam fragments maintain a relatively poor native-like fold in aqueous buffer and are unable to bind Ship2-Sam with high affinity and specificity. However, careful analysis of obtained results along with inspection of the EphA2-Sam sequence, surrounding its binding interface for Ship2-Sam, led us to identify a “KRIAY” motif, made up of amino acids that could provide important interactions with the negatively charged ML surface. This penta-amino acid motif if repeated three times in tandem (=(KRI)_3_ peptide in Table S1) produces appreciable binding to Ship2-Sam. Preliminary in cell studies show that the (KRI)_3_ peptide is endowed with higher cytotoxicity against PC-3 prostate carcinoma cell line, in which endogenous levels of both EphA2 and Ship2 are elevated, compared to normal fibroblasts. The peptide contains a “KRI” three amino-acid sequence that is common to several antibacterial and antimicrobial peptides provided with anticancer activity^38,41^. Additional cell-based investigations are needed to clarify the mechanism of action of the (KRI)_3_ peptide in cancer cells and to establish its correlation to the EphA2/Ship2 signaling. Although the (KRI)_3_ peptide shows some cytotoxicity in normal cells we envision that an increase in selectivity towards cancer cells could be achieved through fusion with tumor-homing motifs^34^ or peptide sequences specific for tumor cells over-expressing EphA2 receptor^42,43^.

In conclusion, the (KRI)_3_ peptide represents a “pioneering peptide ligand” of Ship2-Sam, and opens additional opportunities for the rational design of original classes of active peptide antagonists of the Ship2-Sam/EphA2-Sam complex which could modulate EphA2 receptor endocytosis and degradation, lowering its pro-oncogenic signaling.

## Methods

### Peptides

Peptides were purchased from Proteogenix (Schiltigheim, France) with high purity (>95%), N-terminal acetylation and C-terminal amidation (Supplemental Table S1). For cell-based assays the peptides Pep1^20^ and (KRI)_3_ were conjugated to a modified TAT sequence (GRKKRRQRRRPPQGG)^28^ and to a FITC moiety and were amidated at the C-termini (See Table S1 for details).

### NMR experiments

NMR experiments were acquired on a Varian Unity Inova 600 MHz spectrometer equipped with a cold probe. For S13-wt (1.7 mM concentration) a first set of 1D [^1^H] and 2D [^1^H, ^1^H] spectra were recorded at T = 298 K in PBS pH = 7.4 (composition: 10 mM phosphates, 137 mM NaCl, and 2.7 mM KCl, from Sigma-Aldrich, Milan-Italy), 600 μL sample total volume, 10% (v/v) D_2_O (98% D, Armar Scientific, Switzerland). Additional NMR experiments were conducted by dissolving the peptide (830 μM concentration) in 600 μL of a mixture 2,2,2-trifluoroethanol-d3 (TFE, 99.5% isotopic purity, Sigma-Aldrich)/H_2_O (60/40 v/v). The peptides S13-SS, KRI, (KRI)_2_, (KRI)_3_, FITC-TAT-(KRI)_3_ were first dissolved in PBS at pH = 7.4 at concentrations equal to 1.2, 1.2, 1.5, 1.0, 0.29 mM respectively. NMR spectra were also recorded for (KRI)_3_ at 500 μΜ concentration in presence of 50% v/v TFE. The set of 2D [^1^H, ^1^H] experiments, that were recorded and analyzed for each peptide under the different experimental conditions (as indicated above), included: TOCSY (Total Correlation Spectroscopy)^44^, NOESY (Nuclear Overhauser Enhancement Spectroscopy)^45^, and DQFCOSY (Double Quantum-Filtered Correlated Spectroscopy)^46^. The following acquisition parameters were generally implemented for 2D spectra: 16–64 scans, 128–256 FIDs in t1, 1024 or 2048 data points in t2, TOCSY mixing time equal to 70 ms, NOESY mixing times equal to 200 and 300 ms. *Excitation Sculpting* ^47^ was used for water suppression. The process of proton resonance assignments was carried out with the standard Wüthrich protocol^48^. Chemical shifts were referenced to internal TSP (Trimethylsilyl-3-propionic acid sodium salt-D4, 99% D, Armar Scientific, Switzerland) (0.0 ppm).

Spectra were processed with VNMRJ 1.1D (Varian, Italy) and analyzed with the software NEASY^49^ included in CARA (http://cara.nmr.ch/doku.php/).

The procedure suggested by Kjaergaard and collaborators was used to estimate chemical shift deviations from random coil values for Hα protons (CSD) (http://www1.bio.ku.dk/english/research/bms/research/sbinlab/groups/mak/randomcoil/script/)^50^.

Helical populations were obtained with the equation:

$$[{{\rm{\Delta }}\delta }_{{\rm{H}}{\rm{a}}{\rm{l}}{\rm{p}}{\rm{h}}{\rm{a}} \mbox{-} {\rm{a}}{\rm{v}}{\rm{e}}{\rm{r}}{\rm{a}}{\rm{g}}{\rm{e}}}/(-0.39)]\times 100$$

where [(CSD = Δδ_Hα_ = δ_Hαobserved_ − δ_Ηαrandom-coil_)] was averaged over residues in a helical conformation^22^.

### Structure calculations and analysis

The NMR solution structures of S13-wt in TFE/H_2_O (60/40 v/v) and of (KRI)_3_ in TFE/PBS (50/50 v/v) were calculated using CYANA 2.1^51^. Distance constraints were obtained from 2D [^1^H, ^1^H] NOESY spectra (300 ms mixing time), and angular constraints were generated with the GRIDSEARCH module of CYANA. Structure calculations were initiated from 100 random conformers; the 20 structures provided with the lowest CYANA target functions were additionally validated with the programs MOLMOL^52^ and iCING^53^. The S13-wt structures were deposited in the PDB Database under accession code: 5NZ9.

### NMR interaction studies

To monitor peptide binding to Ship2-Sam chemical shift perturbation studies were conducted by means of 2D [^1^H,^15^N] HSQC experiments^24^. In detail, [^1^H,^15^N] HSQC spectra were acquired for ^15^N labeled Ship2-Sam (concentration 25 µM) in absence and presence of a) S13-wt (870 µM concentration), b) S13-SS (1.1 mM concentration); c) KRI (1 mM concentration), d) (KRI)_2_ (1 mM concentration), for these binding experiments peptides were all dissolved in protein buffer (i.e., PBS pH = 7.4).

Binding of the peptide (KRI)_3_ to Ship2-Sam was investigated by recording several 2D [^1^H, ^15^N] HSQC spectra of ^15^N labeled Ship2-Sam at a fixed concentration (=40 µM) in absence and presence of increasing amounts of peptide (i.e., 80, 152, 300, 586, 1100, 2000 μM). During NMR titrations the pH was checked at each point and eventually adjusted to 7.4 with addition of small aliquots of a concentrated NaOH solution. A NOESY spectrum (300 ms mixing time) was as well recorded for (KRI)_3_ (concentrations equal to 800 µM) after addition of Ship2-Sam (40 µM concentration).

### Displacement experiment

To assess if (KRI)_3_ could compete with EphA2-Sam for the same binding site on the surface of Ship2-Sam, we performed a displacement experiment through 2D [^1^H, ^15^N]-HSQC spectra^15,25,26^. We first recorded a spectrum of ^15^N labeled EphA2-Sam in its free form then, unlabeled Ship2-Sam was added to the sample and consequent changes in the HSQC were revealed. Finally, we tried to restore the spectrum of unbound EphA2-Sam by addition of (KRI)_3_ (EphA2-Sam concentration equal to 25 μM, Ship2-Sam concentration equal to 80 μM, peptide concentrations equal to 1.1 mM, 2.2 mM, 4.9 mM, and 7 mM).

### Circular Dichroism

CD spectra were recorded in a 0.1 cm path-length quartz cuvette on a Jasco J-810 spectropolarimeter (JASCO Corp, Milan, Italy) as already described^20,23^. Spectra derived from the average of three scans, the subtraction of blanks and the conversion of the signal to mean residue ellipticity (deg × cm^2^ × dmol^−1^ × res^−1^). All peptides were analyzed at 100 μM, in 10 mM phosphate at pH = 7.2 and for (KRI)_3_ and S13-wt TFE titrations were carried out as well. CD spectra of (KRI)_3_ were also recorded in presence of Ship2-Sam at different peptide_protein ratios (See Supplemental Material for details).

### SPR

SPR experiments were conducted as previously reported^23^. Ship2-Sam domain was efficiently immobilized by employing a solution at a concentration equal to 25 μg/mL in acetate buffer (10 mM and pH = 5.0), at a flow-rate of 5 μL/min and an injection time of 7 min, on a CM5 sensor chip through amine-coupling procedure; the immobilization level was 2065 RU.

SPR assays were conducted with the following parameters: (i) flow: 20 μL/min, (ii) contact time: 4.5 min; (iii) running buffer: HBS (HEPES (10 mM), NaCl (150 mM), EDTA (3 mM), pH 7.4).

Reference channel signals were subtracted as blanks and the software BIAevaluation (version 4.1, GE Healthcare) was employed to estimate kinetic and thermodynamic parameters of the complexes. The Graph-Pad Prism software (version 7.00; GraphPad Software, San Diego, California) was used to fit RU_max_ for (KRI)_3_ concentrations by nonlinear regression analysis.

### MST

A Monolith NT 115 system (Nano Temper Technologies) with 100% LED and 40% IR-laser power was used to perform MST experiments. The His-Tag Labeling Kit RED-tris-NTA was implemented to achieve protein labeling. For Ship2-Sam labeling, the protein concentration was adjusted to 100 nM in labeling buffer (Nano Temper Technologies)^54^, while the dye concentration was set to 100 nM. Both protein and fluorescent dye solutions were incubated at room temperature in the dark for 30 min. A 16-step serial dilution (1:1) was prepared for (KRI)_3_ (final concentration range 250 μM – 7.6 nM) and premium capillaries were filled with samples. Measurements were conducted at 25 °C in PBS supplemented with 0.05% Tween-20. An equation implemented by the software MO-S002 MO Affinity Analysis, provided by the manufacturer, was used for fitting normalized fluorescence values at different (KRI)_3_ concentrations.

### Protein expression and purification

Recombinant EphA2-Sam and Ship2-Sam were expressed in *E. coli*. Protein constructs along with expression and purification methodologies have been reported in our previous publications^11,20,23^. Proteins were either produced in LB (Luria-Bertani) broth or M9 minimal medium supplemented with 1 g/L of ^15^NH_4_Cl. Bacteria were grown at 37 °C till a cell optical density OD_600nm_ equal to 0.6, then in the induction step isopropyl β-D-1-thiogalactopyranoside (IPTG) at 1 mM concentration (25 °C, overnight) was used. Purification of his-tagged Ship2-Sam and EphA2-Sam was achieved by affinity chromatography with an Akta Purifier FPLC System (GE Healthcare, Milano, Italy) and a Nickel column. Finally, proteins were dialyzed in PBS pH = 7.4.

### Cell culture and cytotoxicity

Human prostate cancer cell lines (PC-3) were cultured in RPMI 1640 medium supplemented with 10% fetal bovine serum (FBS) (GIBCO, USA), 2 mM L-glutamine (Lonza, Belgium) in 5% CO2 humidified atmosphere and harvested at approximately 90% confluence. The normal human dermal fibroblasts (NHDF) were purchased from Lonza, seeded on T-25 primary flasks (Beckton Dickinson) and maintained in fibroblast basal medium (FBM) supplemented with 2% FBS, 5 mg/ml rh insulin, 1 mg/ml hydrocortisone, 50 mg/ml ascorbic acid, 5 ng/ml rh FGF b. For cytotoxicity assay 4 × 10^3^ and 3.5 × 10^3^ were seeded for cancer and normal cells respectively in 50 µl medium per well in 96-well flat-bottom microplates and incubated overnight to allow cell adhesion. Subsequently, culture medium was removed and cells were incubated with 100 µl of their growth medium with different concentrations of peptide in quadruplicate for 4 hours. Reduction of cell viability was determined by the crystal violet assay. Shortly, after treatment, culture medium was removed, cells were washed with phosphate-buffered saline (PBS), then they were fixed and stained with 0.1% (w/v) crystal violet in 25% methanol for 30 minutes at dark; afterwards, crystal violet was removed and cells were washed twice with double distilled water and let dry. In the end, the cells were solubilized by adding 10% (v/v) acetic acid and the amount of dye taken up was quantified with a plate reader (Multiskan Fc 10094, Thermo) at 595 nm. Data were expressed as mean ± SD of three independent experiments.

### Flow cytometer analysis

PC-3 cells, plated at a density of 6 × 10^5^, were incubated with FITC-TAT-peptides for 4 hours at 37 °C. Then, the cells were gently washed twice with PBS, permeabilized with 0.1% Triton X-100 in 33 mM sodium citrate and analyzed with a flow cytometer equipped with a 488 nm argon laser (FACScan, Becton Dickinson, CA, USA). A total of 20,000 events per sample were collected. Values of fluorescence intensity were obtained from histogram statistic of CellQuest software.

### LDH assay

The release of LDH was used as a marker for cytotoxicity. After treatment with 50 µM FITC-TAT-(KRI)_3_ for 4 hours, cleared supernatants were incubated with reaction buffer for 30 minutes at room temperature according to the manufacturer instructions (ab65393, LDH-Cytotoxicity Assay Kit II). Cells untreated and treated with 1% Triton-X 100 in PBS were used respectively as negative and positive control. Absorbance was measured at 490 nm and the cytotoxicity percentage was calculated as: [(Absorbance_test sample – Absorbance_low control)/(Absorbance_high control – Absorbance_low control) × 100.

### Data availability statement

S13-wt NMR structures in presence of TFE have been deposited in the Protein Data Bank (PDB entry code: 5NZ9). NMR structures calculated in presence of TFE for (KRI)_3_, whose amino acid sequence is short and unnatural, are not publicly available but will be provided by the corresponding author on reasonable request.

## Electronic supplementary material

Supporting Information
