# Supplementary material for: The Sam-Sam interaction between Ship2 and the EphA2 receptor: design and analysis of peptide inhibitors
Source: Sci Rep. 2017 Dec 12;7:17474. doi: 10.1038/s41598-017-17684-5 (PMC5727260; doi:10.1038/s41598-017-17684-5)
Supplement: Supplementary file 1 — Supporting Information [file 41598_2017_17684_MOESM1_ESM.pdf]

The Sam-Sam interaction between Ship2 and the EphA2 receptor: design and analysis of peptide inhibitors

Flavia Anna Mercurio[1], Concetta Di Natale[2], Luciano Pirone[1], Roberta Iannitti[1], Daniela Marasco[1,2], Emilia Maria Pedone[1], Rosanna Palumbo[1], and Marilisa Leone[1*]

[1]Institute of Biostructures and Bioimaging (IBB), CNR, via Mezzocannone 16, 80134-Naples, Italy

[2]Department of Pharmacy, Research Centre on Bioactive Peptides (CIRPeB), University of Naples "Federico II", Via Mezzocannone 16, 80134-Naples, Italy

*Corresponding Author:
Marilisa Leone
Institute of Biostructures and Bioimaging (CNR), Via Mezzocannone, 16 – 80134 Naples, Italy.
Tel: +39-081-2534512
E-mail address: marilisa.leone@cnr.it.

Table S1. Peptide sequences analyzed in the manuscript.

	Peptide sequence
**S13-wt**	Ac-KRIGVRLPGHQKRIAYSLLGLKDQV-NH_2
**S13-SS**	Ac-KRIGVRLPGHQKR-C-AYSLLGLKDQV-NH_2<br>| Ac-S-C-KMQQ-NH_2
**KRI**	Ac-GHQKRIAY-NH_2
**$(KRI)_2$**	Ac-KRIAYKRIAY-NH_2
**$(KRI)_3$**	Ac-KRIAYKRIAYKRIAY-NH_2
**FITC-TAT-$(KRI)_3$**	{FITC}-{β-Ala}-GRKKRRQRRRPPQGG-KRIAYKRIAYKRIAY-NH_2
**FITC-TAT-Pep1**	{FITC}-Ahx*-{β-Ala}-GRKKRRQRRRPPQGG-<br>SKLLLNGFDDVHFLGSNVMEEQ-NH_2

*AhX=aminohexanoic linker
Ac=N-terminal acetylation
-NH_2=C-terminal amidation

Table S2. Chemical shifts of S13-wt peptide in PBS pH 7.4 and T=25°C.

Residue	H_N	Hα	Hβ	Hγ	Others
**1K**					Acetyl 2.05
**2R**	8.15	4.40	1.80-1.83	1.60-1.66	Hδ 3.20
**3I**	8.23	4.21	1.89	1.23-1.50<br>CH3γ 0.93	δCH3<br>0.87
**4G**	8.50	3.95-3.99			
**5V**	7.94	4.12	2.05	0.92	
**6R**	8.43	4.38	1.75-1.79	1.59-1.62	Hδ 3.16<br>Hε 7.00
**7L**	8.40	4.62	1.54-1.65		δCH3<br>0.91
**8P**		4.42	1.91-2.33	2.05	Hδδ'<br>3.67-3.85
**9G**					
**10H**	8.11	4.58	3.14		Hδ 7.00<br>Hε 7.81
**11Q**		4.30	1.95-2.07	2.31	
**12K**					
**13R**	8.34	4.34	1.82	1.61	Hδ 3.19
**14I**	8.15	4.09	1.79	1.16-1.45<br>CH3γ 0.80	δCH3<br>0.84
**15A**	8.29	4.32	1.35		
**16Y**	8.09	4.53	2.99-3.05		Hδ 7.13<br>Hε 6.84
**17S**	8.19	4.41	3.83-3.88		
**18L**	8.23	4.36	1.67		δCH3<br>0.88-0.94
**19L**	8.01	4.32	1.61-1.68		δCH3<br>0.87-0.93
**20G**	8.30	3.92-3.95			
**21L**	7.96	4.34	1.61-1.66		δCH3<br>0.87-0.94
**22K**	8.32	4.29	1.81-1.84	1.45	Hδ 1.69<br>Hε 3.01
**23D**	8.23	4.58	2.64-2.70		
**24Q**	8.22	4.36	2.00-2.16	2.37	Hε 6.86–7.54
**25V**	8.12	4.12	2.10	0.96	

Table S3. Chemical shifts of $(KRI)_3$ peptide in TFE/PBS 50/50 v/v at pH 7.1 and T=25°C.

Residue	HN	Hα	Hβ	Hγ	Others
**1K**	8.24	4.13	1.82	1.47-1.55	Hδ 1.76<br>Hε 3.04<br>Acetyl 2.12
**2R**		4.25	1.92	1.72-1.81	Hδ 3.26
**3I**	7.61	4.01	1.96	1.24-1.58<br>CH3γ 0.94	δCH3<br>0.92
**4A**	7.93	4.19	1.46		
**5Y**	8.04	4.33	3.14		Hδ 7.12<br>Hε 6.86
**6K**	7.89	4.05	1.95	1.47-1.61	Hδ 1.73<br>Hε 3.02
**7R**	8.07	4.19	1.98	1.72-1.83	Hδ 3.18
**8I**	7.94	3.89	1.94	1.19-1.73<br>CH3γ 0.95	δCH3<br>0.90
**9A**	8.10	4.08	1.41		
**10Y**	8.09	4.31	3.15-3.18		Hδ 7.13<br>Hε 6.85
**11K**	7.94	4.05	1.97	1.49-1.63	Hδ 1.75<br>Hε 3.00
**12R**	7.99	4.17	2.00	1.71-1.88	Hδ 3.22
**13I**	7.94	3.96	1.90	1.18-1.64<br>CH3γ 0.91	δCH3<br>0.87
**14A**	8.10	4.10	1.15		
**15Y**	7.79	4.53	3.01-3.19		Hδ 7.13<br>Hε 6.84<br>$CONH_2$<br>7.21-6.87

Table S4. Structure statistics for S13-wt peptide solution structure in TFE/H_2O 60/40 v/v.

**Residual NOE violations**	3
Number ≥ 0.1 **Å***	0
**Residual angle violations**	0
**Atomic pairwise RMSD, Å**	
Backbone atoms (aa 10-23)	0.24±0.08
Heavy atoms (aa 10-23)	1.06±0.15
**Procheck analysis (all residues)**[#]	
Residues in core regions	81.8%
Residues in allowed regions	18.2%
Residues in generous regions	0.0%
Residues in disallowed regions	0.0%

[*] CYANA[1] Mean violations
[#]PROCHECK_NMR[2]statistics.

Table S5. Structure statistics for $(KRI)_3$ peptide solution structure in TFE/PBS 50/50 v/v.

**Residual NOE violations**	0
**Residual angle violations**	0
**Atomic pairwise RMSD, Å**	
Backbone atoms (all residues)	0.38±0.11
Heavy atoms (all residues)	1.28±0.13
**Procheck analysis (all residues)#**	
Residues in core regions	81.9%
Residues in allowed regions	18.1%
Residues in generous regions	0.0%
Residues in disallowed regions	0.0%

* CYANA[1] Mean violations
#PROCHECK_NMR[2] statistics.

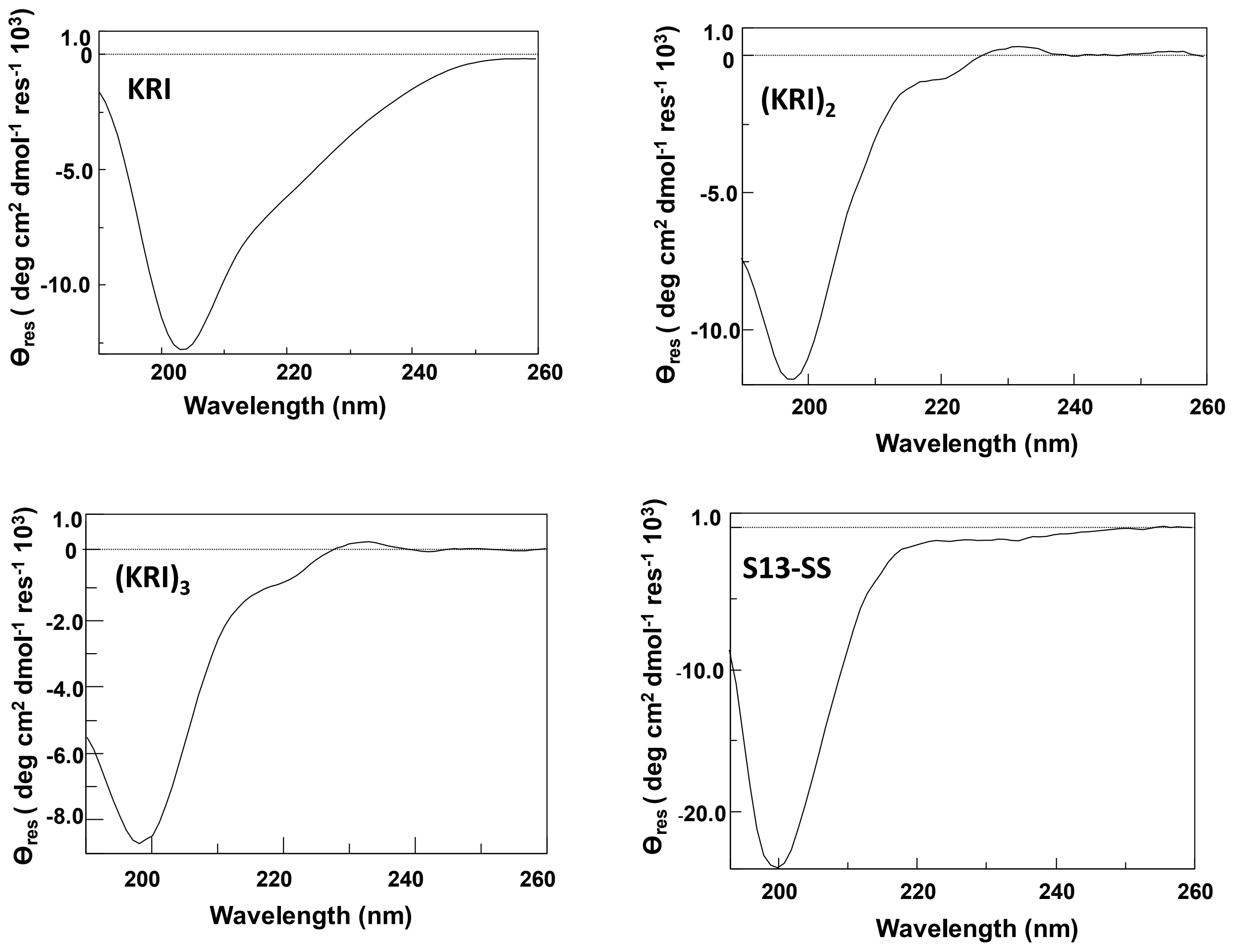

Fig. S1. CD spectra of KRI, $(KRI)_2$, $(KRI)_3$, S13-SS (100 µM concentration each), recorded in 10 mM sodium phosphate pH 7.2.

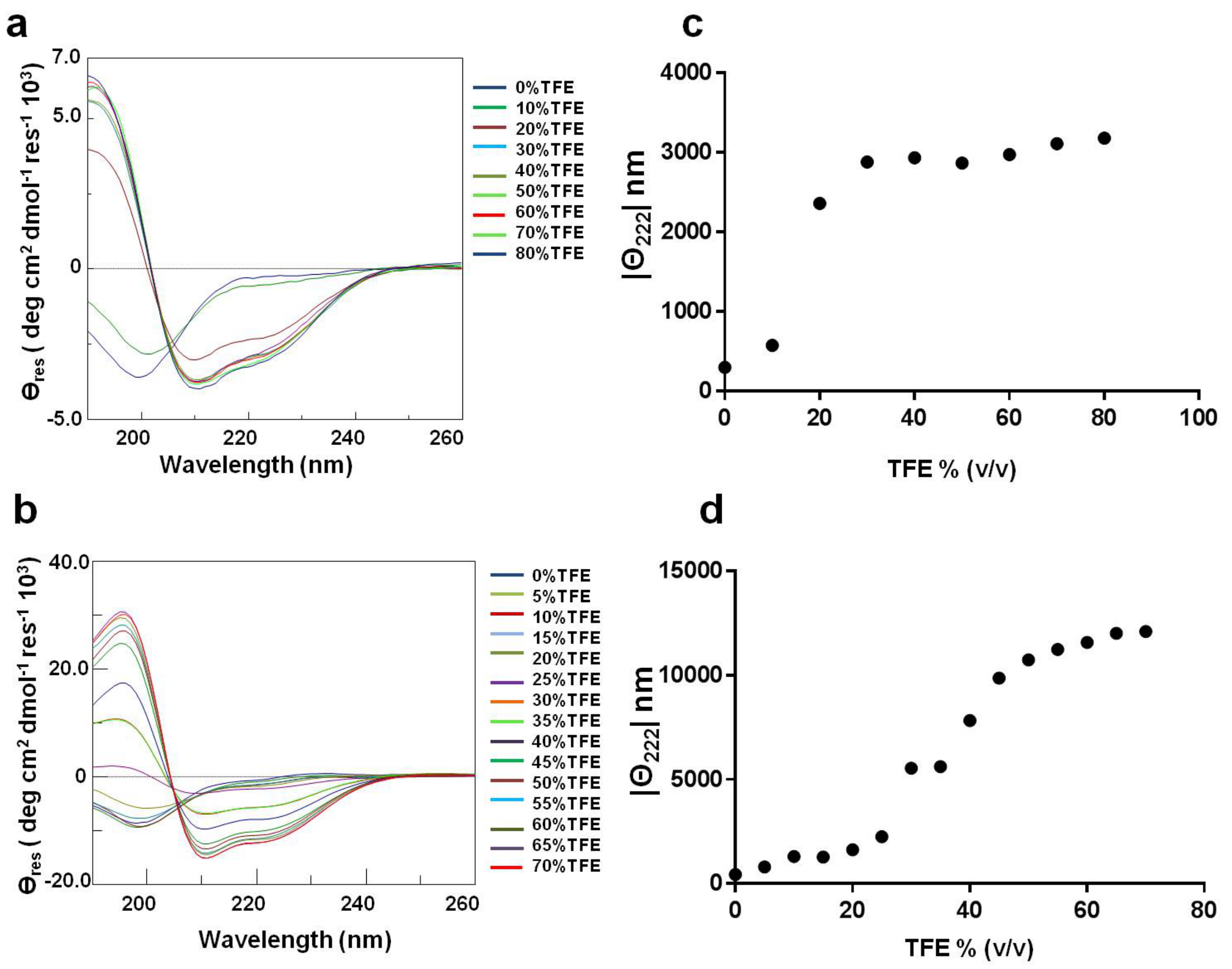

Fig. S2. (a, b) Overlay of CD spectra recorded at different TFE % (v/v) for S13-wt (a) and $(KRI)_3$ (b). The shift of λ minimum toward 210 nm, the appearance of a new relative minimum around 222 nm and maximum at 190 nm, upon increasing the amount of TFE, clearly indicate intensification of helical content. (c, d) Absolute values of Θ_{222} nm *versus* increasing amounts of TFE for S13-wt (c) and $(KRI)_3$ (d).

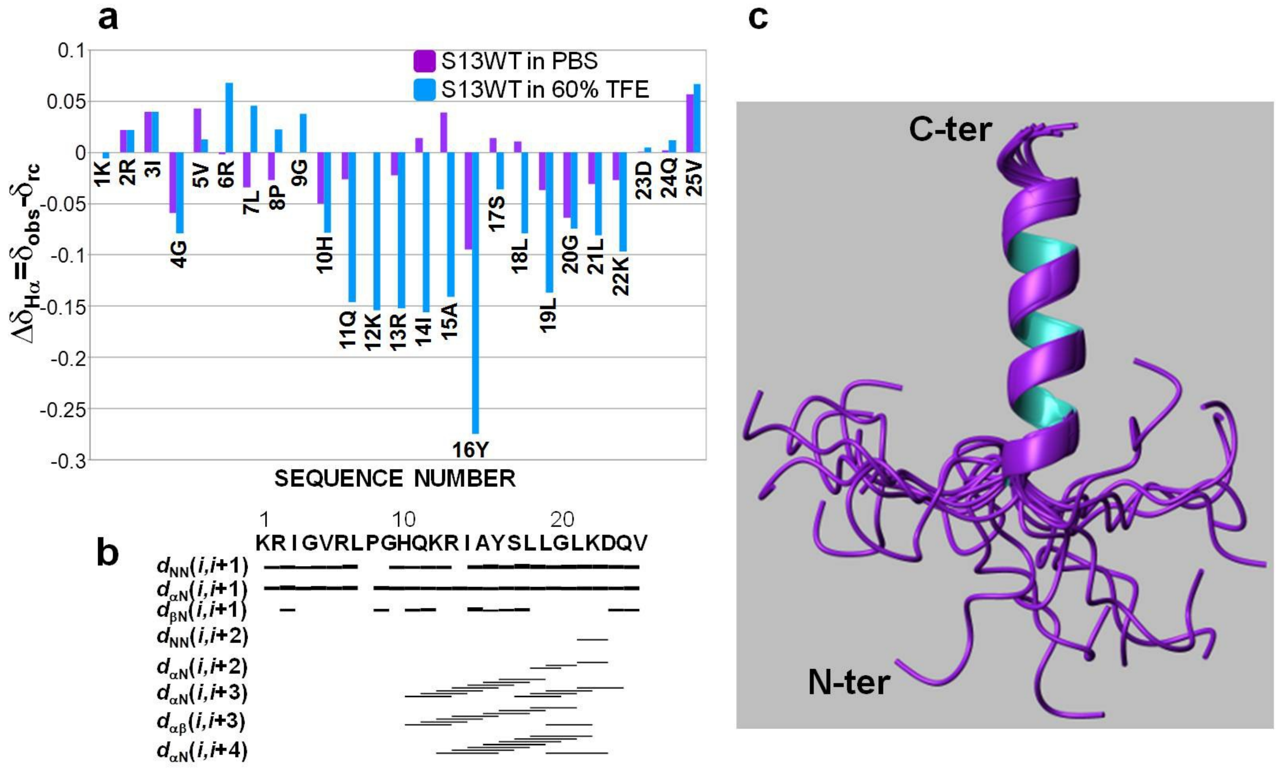

Fig. S3. (a) Chemical shift deviations of S13-wt peptide Hα protons from random coil values calculated in TFE/H_2O 60/40 v/v (light blue), and in PBS (violet). Random coil chemical shifts refer to 298 K and pH=7.4 (sample in PBS) or pH=3.5 (sample in TFE/H_2O 60/40 v/v). (b) S13-wt peptide NOEs pattern in 60% TFE; "dxy*(i, i+1)*" stands for a NOE correlation between protons x and y in the i and i+1 residues respectively; the thickness of each bar is proportional to the corresponding NOE intensity. (c) Ribbon representation of S13-wt NMR ensemble of structures obtained in presence of 60% TFE. The final structure calculation includes 224 distance restraints (86 intrarresidue, 94 short- and 44 medium-range) along with 127 angle constraints. In the Figure the best twenty S13-wt NMR conformers with lowest target function values, are superimposed on the backbone atoms of residues from 10 to 23. Structures have been deposited in the Protein Data Bank (PDB entry code: 5NZ9).

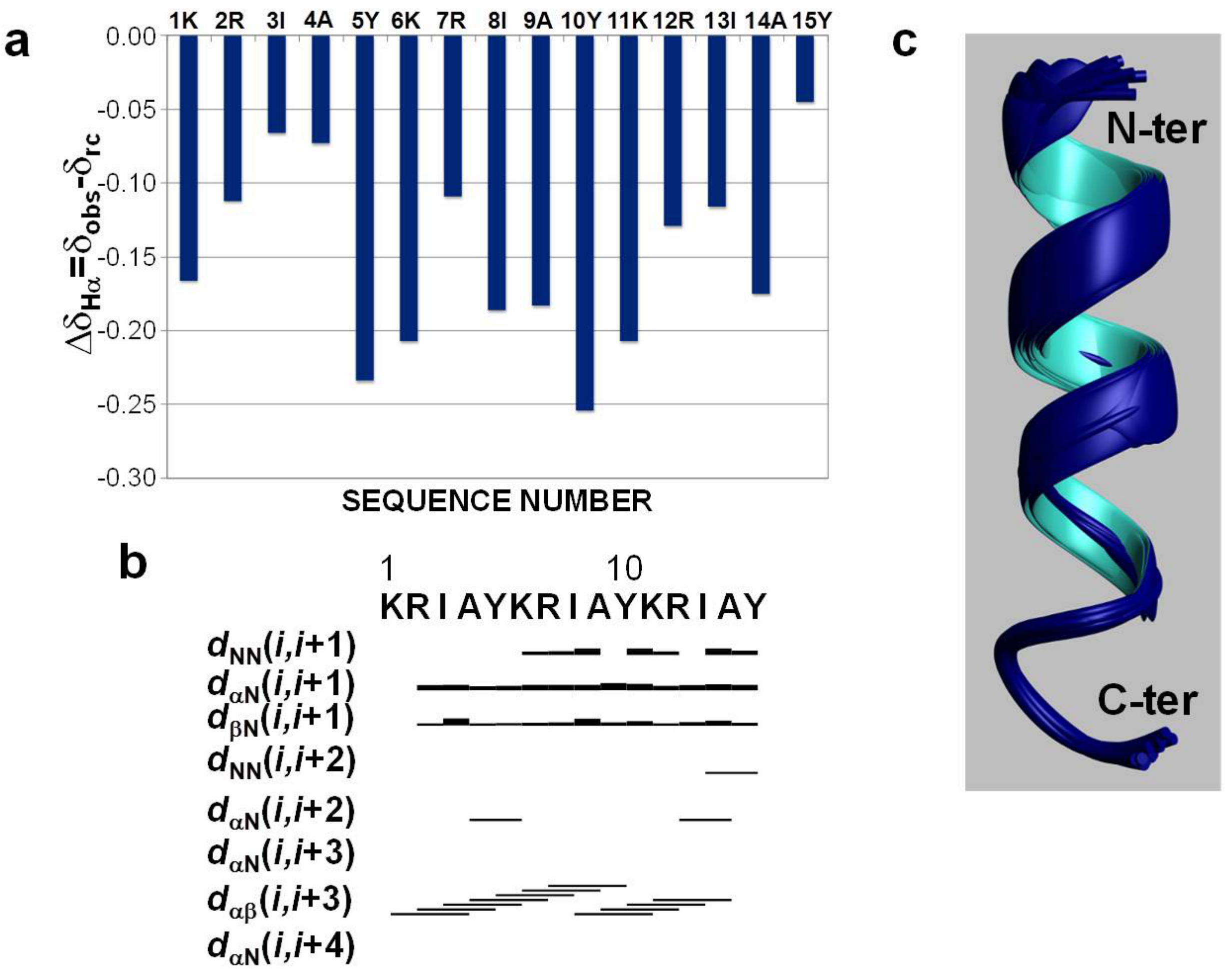

Fig. S4. NMR analysis of the $(KRI)_3$ peptide in TFE/PBS 50/50 v/v. (a) Chemical shift deviations of Hα protons from random coil values. Random coil chemical shifts were estimated as indicated in the Methods section at T=298 K and pH=7. (b) NOEs pattern. (c) Ribbon representation of the best twenty $(KRI)_3$ NMR structures superimposed on the backbone atoms. The final structure calculation includes 131 distance (47 intrarresidue, 39 short-, 43 medium-, 2 long-range) and 83 angle restraints respectively.

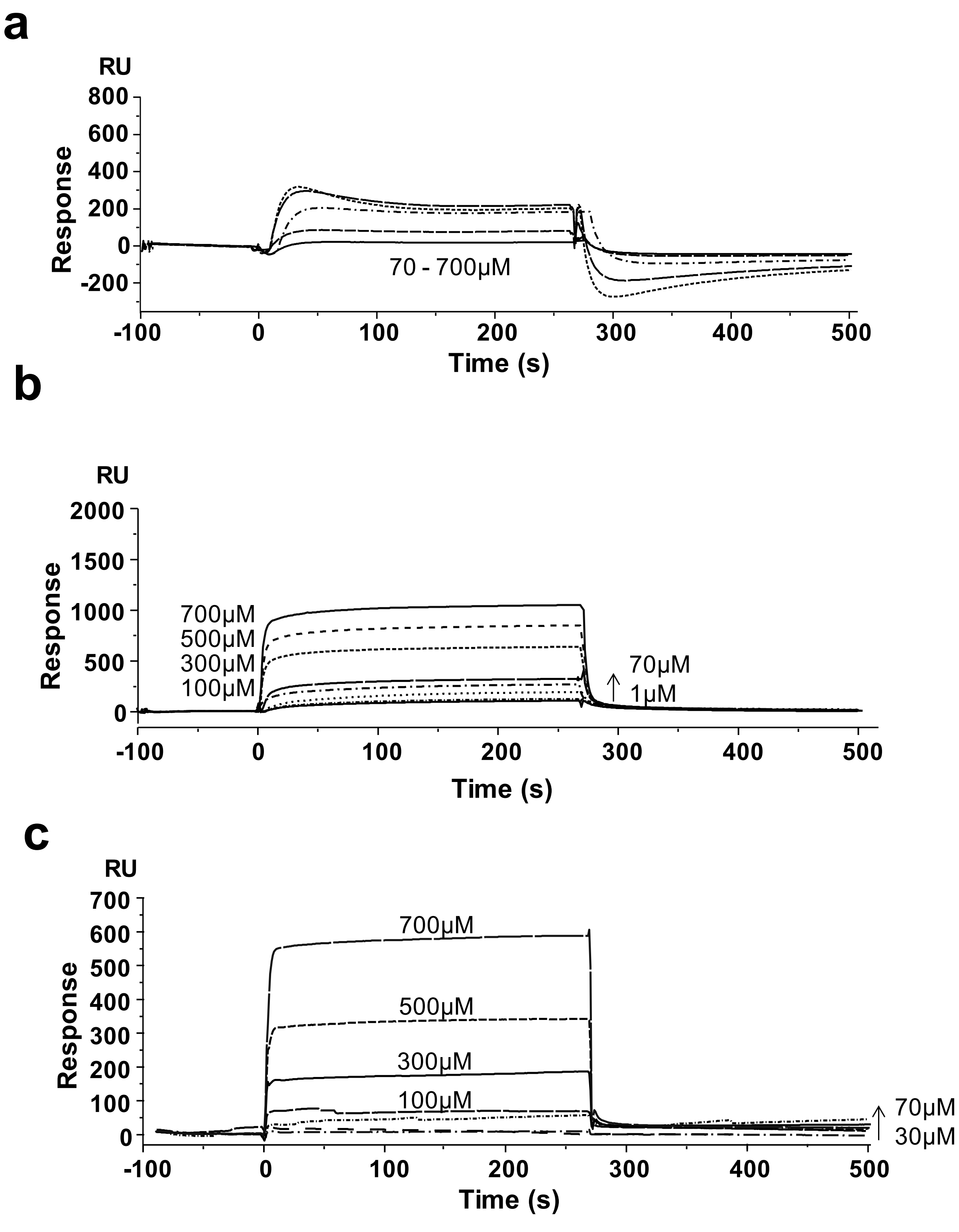

Fig. S5. Sensorgrams relative to the binding of (a) S13-wt; (b) S13-SS and (c) $(KRI)_2$ to immobilized Ship2-Sam.

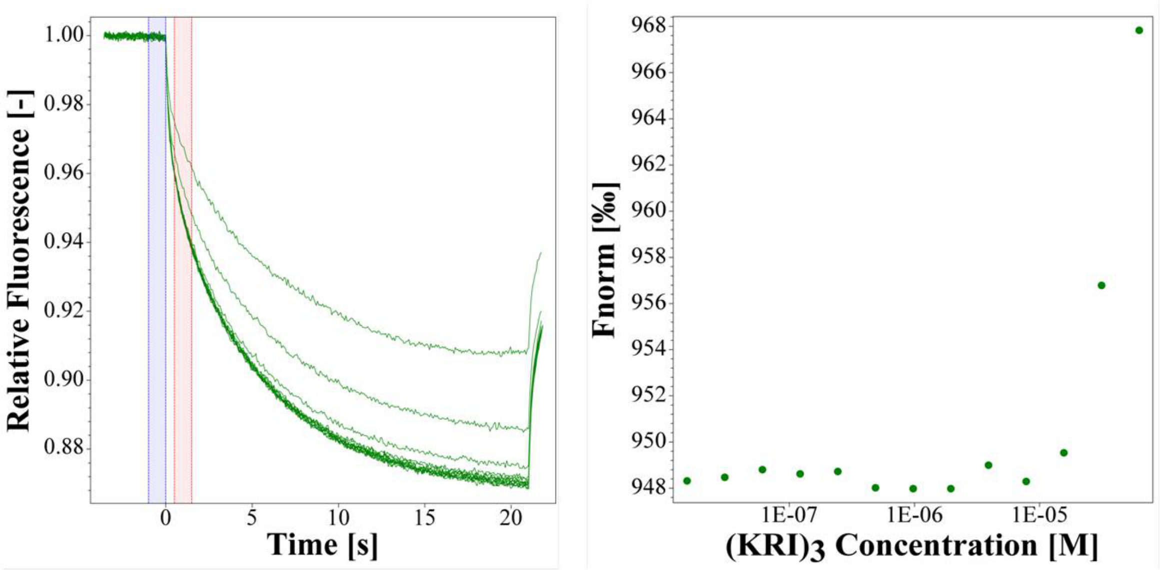

Fig. S6. Binding of $(KRI)_3$ to Ship2-Sam monitored by MST. MST traces for the titration of $(KRI)_3$ against Ship2-Sam and MST signals as function of increasing peptide concentrations are reported in the left and right panels respectively.

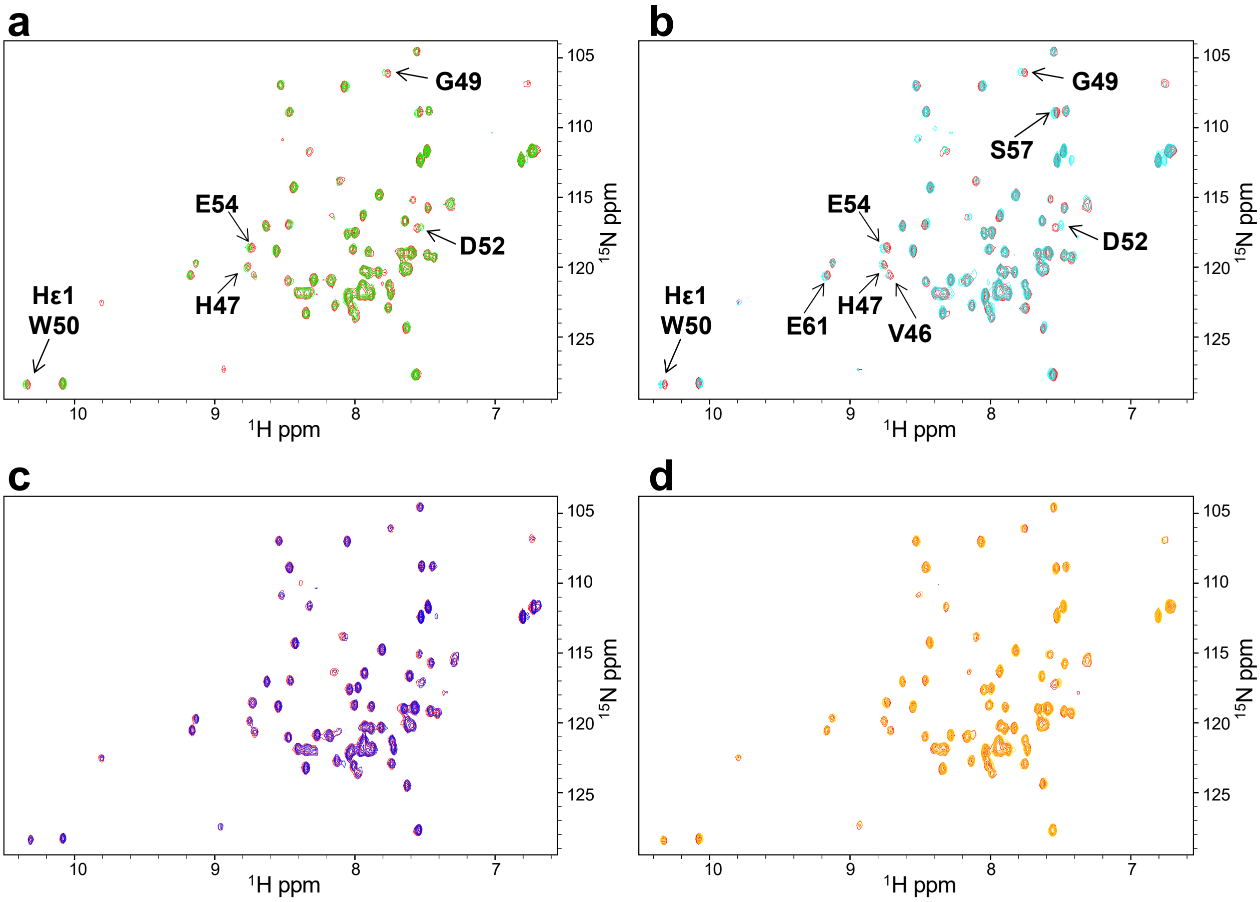

Fig. S7**.** Superposition of 2D [^{1}H-^{15}N] HSQC spectra of Ship2-Sam in its free form (25 µM-red) and in presence of: (a) S13-wt (870 µM concentration-green); (b) S13-SS (1.1 mM concentration-cyan) (c) KRI (1 mM concentration-blue) and (d) $(KRI)_2$ (1 mM concentration-gold). Assignments of a few peaks undergoing small chemical shifts and/or intensity variations upon addition of S13-wt (a) and S13-SS (b) to Ship2-Sam have been indicated.

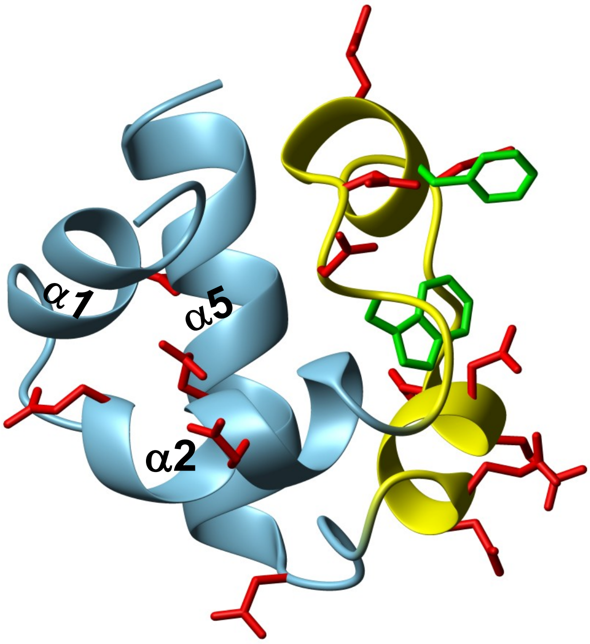

Fig. S8. One representative conformer (number 1) of the Ship2-Sam solution structure (pdb entry 2K4P[3]) in a ribbon representation where side chains of Asp and Glu are shown along with Phe and Trp residues in the ML region.

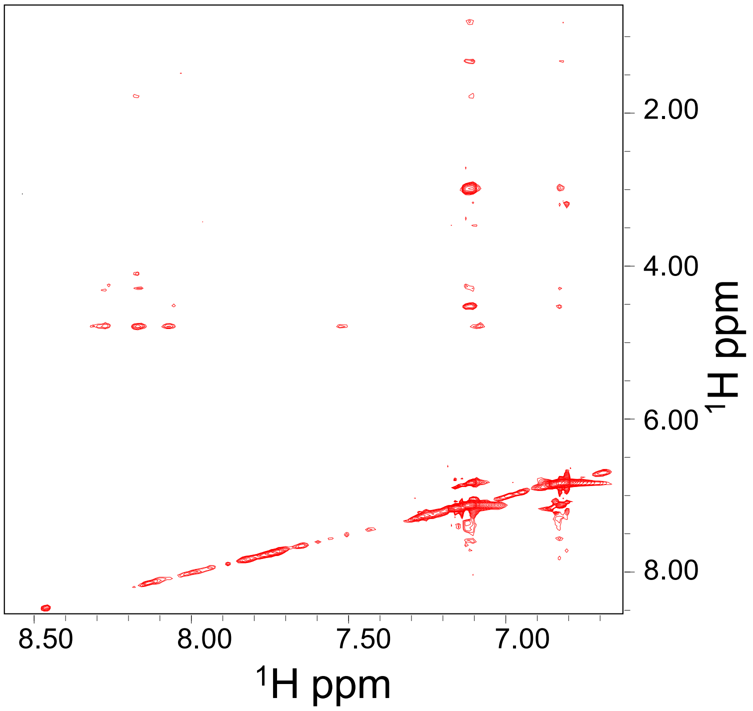

Fig. S9. Expansion of a region from the NOESY 300 spectrum of $(KRI)_3$ (800 µM, in PBS buffer pH=7.4) in presence of a sub-stoichiometric amount of Ship2-Sam (40 µM).

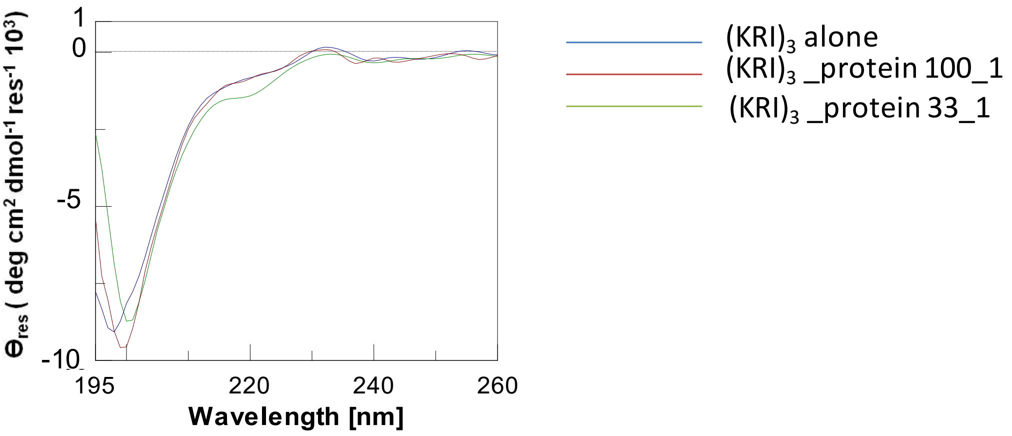

Fig. S10. CD spectra of $(KRI)_3$ (8.33 μM concentration in buffer 10 mM phosphate pH=7.4, cell path 1.0 cm) alone and at different peptide_Ship2-Sam ratios (1_0, 100_1, 33_1). Ship2-Sam concentrations are 0, 0.0833, 0.251 μM, respectively. A slight shift of the wavelength of the minima (from 197 to 201 nm) indicates the presence of more ordered conformations. Higher ratios cannot be explored since the contribution of the protein alone (blank) is greater than in combination with the peptide.

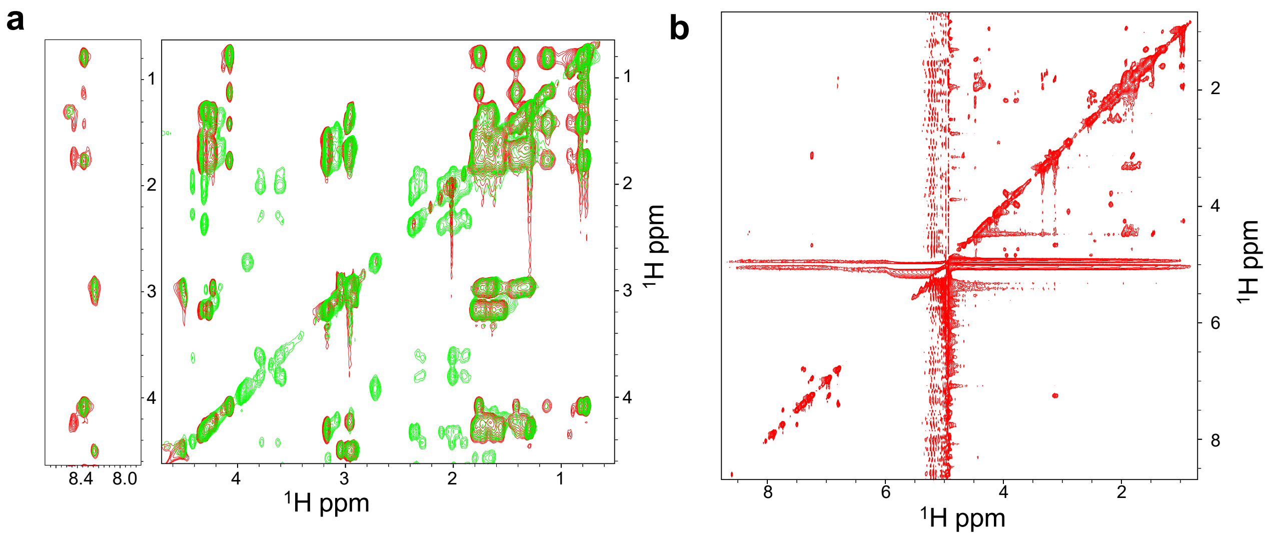

Fig. S11. NMR analysis of the FITC-TAT-(KRI)$_3$ peptide (290 µM concentration in PBS pH=7.4). (a) Superposition of H_N-aliphatic (left panel) and side chains-side chains (right panel) regions of TOCSY spectra acquired for (KRI)$_3$ (1 mM-red) and FITC-TAT-(KRI)$_3$ (green). (b) NOESY 300 spectrum of FITC-TAT-(KRI)$_3$. NMR spectra are characteristic of disordered peptides.

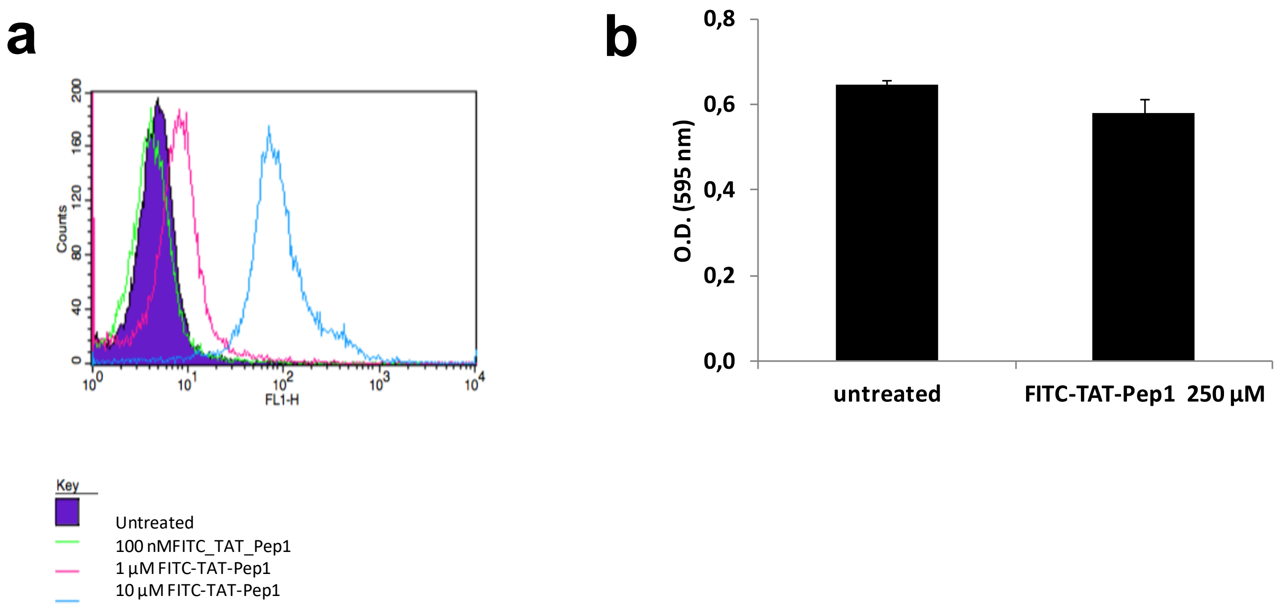

Fig. S12. Internalization and cytotoxicity of FITC-TAT-Pep1. (a) Internalization of the peptide was detected by flow cytometric analysis on permeabilized PC-3 cells after incubation with different amounts of FITC-TAT-Pep1 for 4 hours. Untreated cells were used as negative control. (b) Cytotoxicity of FITC-TAT-Pep1 (250 µM concentration) was assessed on PC-3 cells, after 4 hours the crystal violet assay was performed. Values represent the mean ± SD of quadruplicate data from three independent experiments.

References

1 Herrmann, T., Guntert, P. & Wuthrich, K. Protein NMR structure determination with automated NOE assignment using the new software CANDID and the torsion angle dynamics algorithm DYANA. *J Mol Biol* 319, 209-227, doi:10.1016/S0022-2836(02)00241-3 (2002).

2 Laskowski, R. A., Rullmannn, J. A., MacArthur, M. W., Kaptein, R. & Thornton, J. M. AQUA and PROCHECK-NMR: programs for checking the quality of protein structures solved by NMR. *J Biomol NMR* **8**, 477-486, doi: 10.1007/BF00228148 (1996).

3 Leone, M., Cellitti, J. & Pellecchia, M. NMR studies of a heterotypic Sam-Sam domain association: the interaction between the lipid phosphatase Ship2 and the EphA2 receptor. *Biochemistry* **47**, 12721-12728, doi:10.1021/bi801713f (2008).
